# Early Disruption of the Microbiome Leading to Decreased Antioxidant Capacity and Epigenetic Changes: Implications for the Rise in Autism

**DOI:** 10.3389/fncel.2018.00256

**Published:** 2018-08-15

**Authors:** Rebecca S. Eshraghi, Richard C. Deth, Rahul Mittal, Mayank Aranke, Sae-In S. Kay, Baharak Moshiree, Adrien A. Eshraghi

**Affiliations:** ^1^Division of Gastroenterology, Department of Medicine, Miller School of Medicine, University of Miami, Miami, FL, United States; ^2^Department of Pharmaceutical Sciences, College of Pharmacy, Nova Southeastern University, Fort Lauderdale, FL, United States; ^3^Department of Otolaryngology, Miller School of Medicine, University of Miami, Miami, FL, United States; ^4^Dr. Kiran C. Patel College of Osteopathic Medicine, Nova Southeastern University, Fort Lauderdale, FL, United States

**Keywords:** autism, oxidative stress, epigenetics, gut microbiota, dysbiosis

## Abstract

Currently, 1 out of every 59 children in the United States is diagnosed with autism. While initial research to find the possible causes for autism were mostly focused on the genome, more recent studies indicate a significant role for epigenetic regulation of gene expression and the microbiome. In this review article, we examine the connections between early disruption of the developing microbiome and gastrointestinal tract function, with particular regard to susceptibility to autism. The biological mechanisms that accompany individuals with autism are reviewed in this manuscript including immune system dysregulation, inflammation, oxidative stress, metabolic and methylation abnormalities as well as gastrointestinal distress. We propose that these autism-associated biological mechanisms may be caused and/or sustained by dysbiosis, an alteration to the composition of resident commensal communities relative to the community found in healthy individuals and its redox and epigenetic consequences, changes that in part can be due to early use and over-use of antibiotics across generations. Further studies are warranted to clarify the contribution of oxidative stress and gut microbiome in the pathophysiology of autism. A better understanding of the microbiome and gastrointestinal tract in relation to autism will provide promising new opportunities to develop novel treatment modalities.

## Introduction

**FIGURE 1:**
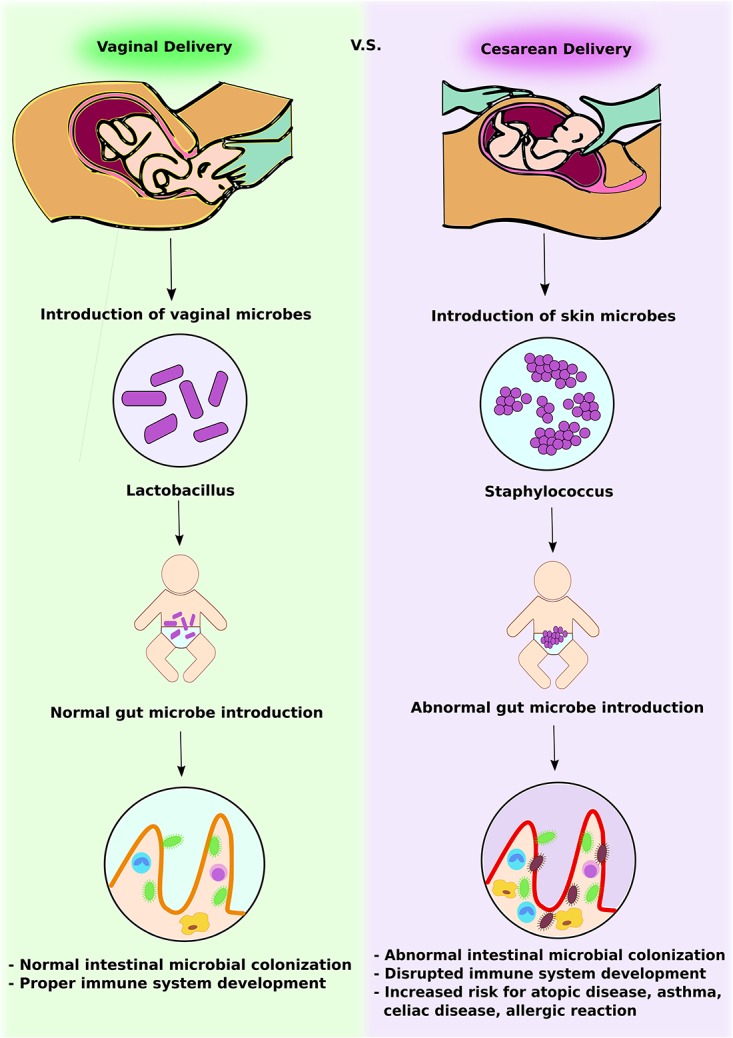
Intestinal microbial introduction by vaginal delivery vs. Cesarean delivery. In vaginal delivery, infants obtain *Lactobacillus* via the vaginal canal. This promotes normal intestinal microbial colonization and development of a competent gut immune system. In contrast, in Cesarean delivery, infants obtain skin microbes, including *Staphylococcus*. This abnormal gut microbe introduction leads to altered intestinal microbial colonization and increases the risk of immunologic disorders.

**FIGURE 2:**
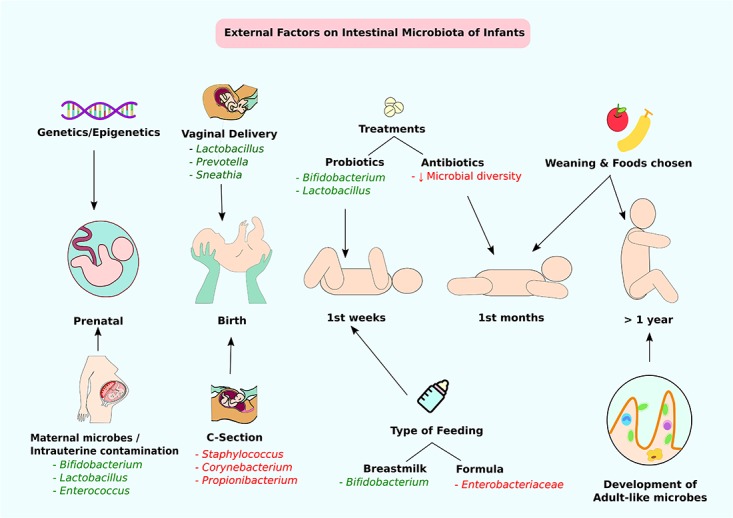
External factors affecting the intestinal microbiota of infants. Through infant developmental stages, multiple factors affect the constitution of intestinal microbiota. Beneficial modifications are highlighted in green and negative alterations are highlighted in red. At the prenatal stage, genetic factors or maternal microbes and intrauterine contamination can affect intestinal colonization. At birth, the delivery method is the main determining factor of gut microbiota. Type of feeding and probiotic/antibiotic treatments at weeks and months can contribute to alteration of intestinal microbes. Approximately at 1 year of age, infants accomplish adult-like gut microbe colonization.

Autism spectrum disorder (ASD) is a pervasive neurodevelopmental disorder characterized by impaired social communication and repetitive as well as stereotyped patterns of behavior. Although autism is mostly recognized by means of behavioral features and autism related disabilities, co-morbid factors such as distinct gut microbial composition, heightened immune response and gastrointestinal abnormalities have been observed (Rose et al., 2018). In addition to these characteristics, alteration in the composition of the gut microbiome has been observed in ASD individuals (Parracho et al., 2005; Finegold et al., 2010; Kang et al., 2018). Multiple factors can change the constitution of the microbiome such as vaginal flora at birth, cesarean section vs. vaginal delivery, breast milk vs. bottle feeding, early nutrition and environmental exposures, as well as antibiotics use, which has one of the most significant effects on microbial ecology (Martin et al., 2016; Bilbo et al., 2017) (**Figures 1**, **2**). Among other consequences, early disruption of a developing gut microbiota can adversely affect antioxidant production (Mardinoglu et al., 2015; Gyuraszova et al., 2017).

Besides antioxidant production, the nature of the microbial community in the gastrointestinal (GI) tract can affect the risk of obesity, diabetes, colon cancer, and autoimmunity (Turnbaugh et al., 2006; Sobhani et al., 2011; Qin et al., 2012; Thursby and Juge, 2017). It is believed that an infant’s gut is sterile until the first exposure to microbes at birth when the baby passes through the birth canal and gets coated with microbes from the mother, or swallows microbes from the mother’s vagina (Macpherson et al., 2017). After these initial exposures, the infant is continuously exposed to bacteria through the mother’s breast milk, food consumption and the environment (Jost et al., 2013; Chu et al., 2016; Gregory et al., 2016). Over time the microbiome becomes individualized, although the composition is strongly dependent on parents and even ancestors (Gibiino et al., 2017). No two individuals share the same microbiome composition, not even identical twins (Turnbaugh et al., 2009).

The GI microbiota can play a crucial role in bidirectional communication between the gut and the brain (Mittal et al., 2017; de la Fuente-Nunez et al., 2018). The gut microbiota influences brain function through the neuroendocrine, neuroimmune and autonomic nervous systems (Kim et al., 2018; Sylvia and Demas, 2018). The aim of this article is to review recent advancements in understanding how the disruption of gut microbiota can alter antioxidant homeostasis in the GI tract and how that can increase the risk of developing autism.

## Biological Mechanisms Associated With Autism

### Immune System Abnormalities

The gut microbiota plays an important role in shaping host immunity. Gut dysbiosis has been associated with immune system abnormalities and has been implicated in the pathogenesis of inflammatory diseases such as systemic lupus erythematosus (SLE) and rheumatoid arthritis (RE) (Liu et al., 2013; Hevia et al., 2014; Zhang et al., 2015; Luo et al., 2018). In addition to these disorders, gut dysbiosis leading to immune system abnormalities has been hypothesized to play a crucial role in the pathophysiology of ASD.

**FIGURE 3:**
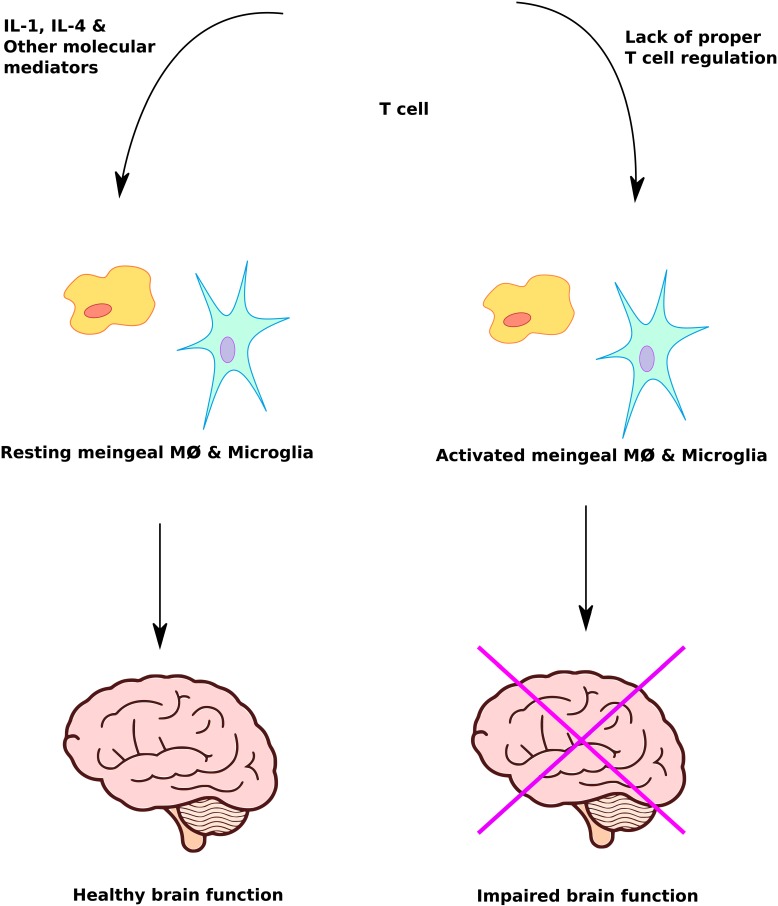
Immune system mediated regulation of brain function. Proper immune system response plays a crucial role in protecting and maximizing brain function. Lack of T cell regulation leads to inappropriate activation of meningeal macrophage and microglia cells, causing impairment of brain function.

Improperly activated immune system abnormalities such as neuroinflammation, pro-inflammatory cytokines, immunoglobulins, immune cellular activation and autoimmunity are significant risk factors in ASD (Goines and Van de Water, 2010; Morgan et al., 2010; Edmiston et al., 2017; Fluegge, 2017; Masi et al., 2017; Wong and Hoeffer, 2017). Dysregulation of T cell responses can lead to activation of meningeal macrophages and glia cells with an adverse effect on brain function (**Figure 3**). A study of post-mortem brain tissues from individuals with ASD demonstrated CNS inflammation, a significantly higher incidence of pro-inflammatory and Th1 cytokines than control group (Morgun et al., 2015). There is evidence of autoimmunity with circulating antibodies directed toward brain proteins in individuals with autism (Li et al., 2009). Finally, cell-mediated immunity is impaired in ASD, as shown by low numbers of CD4 cells and associated T-cell polarity with an imbalance of Th1/Th2 subsets toward Th2 (Filiano et al., 2015). Deviations in the level of natural killer (NK) cells and macrophages have been observed in individuals with autism compared to controls (Cohly and Panja, 2005; Vojdani et al., 2008). Taken together these studies suggest that immune system abnormalities are prevalent in ASD patients. However, further studies using larger sample sizes are required to confirm and understand the role of immune system abnormalities underlying the pathophysiology of ASD.

### Impaired Methylation and Oxidative Stress

In addition to immune system abnormalities, altered DNA methylation patterns have been observed in ASD patients. DNA methylation is a highly complex and dynamic process enabling epigenetic regulation of gene expression (Segen, 2005; Chen et al., 2017; Krejcova et al., 2017; Zhang et al., 2017; Kim et al., 2018; Meehan et al., 2018). Epigenetic regulation provides a mechanism by which the body can adaptively deal with stress as well as infections and toxins. It is a fundamental cellular pathway that supports detoxification while regulating inflammation and balancing the action of neurotransmitters (James et al., 2004; MacDonald and Roskams, 2009; McCall et al., 2010; Berk et al., 2011; Hedrich, 2017; Samanta et al., 2017). It is now apparent that methylation-dependent epigenetic regulation is the fundamental driving force behind development, including both prenatal and postnatal development. Consequently, those functions of the GI tract and its microbiome that support methylation capacity are essential for normal development. Vitamin B12 (cobalamin) and folic acid (folate) are two of the most important nutritional factors for methylation and their efficient absorption is essential for normal epigenetic regulation and postnatal development (DeVilbiss et al., 2015; Athanasopoulos et al., 2016; Crott, 2017). Folate is produced by bacteria for their own metabolic needs, but only certain bacterial strains (e.g., *Bifidobacterium* and *Lactobacillus*) are capable of producing vitamin B12, setting up competition between microbes and intestinal epithelial cells in the distal ileum, the primary site of B12 absorption (Degnan et al., 2014). There are specific transport mechanisms for intestinal uptake of B12 bound to its carrier protein intrinsic factor and pathological GI conditions such as inflammation and/or oxidative stress may adversely affect B12 absorption by their effects on the transport process.

The alteration in genetic composition can also influence methylation and nutritional absorption including vitamin B12 and folic acid absorption. A major genetic determinant of methylation capacity is the presence or absence of single nucleotide polymorphisms (SNPs) in the gene for 5,10-methylenetetrahydrofolate reductase (*MTHFR*) (Yuan et al., 2017). MTHFR catalyzes formation of 5-methyltetrahydrofolate, the source of methyl groups for the B12 and methylfolate-dependent enzyme methionine synthase (MS), which converts homocysteine (HCY) to methionine (MET) (Wang and Jin, 2017). MS activity exerts general control over hundreds of methylation reactions, including DNA methylation, because it increases the level of the universal methyl donor *S*-adenosylmethionine (SAM) while at the same time decreasing the level of *S*-adenosylhomocysteine (SAH), which is an inhibitor of methylation reactions and the precursor molecule to homocysteine in the methionine cycle. There are two relatively common SNPs in *MTHFR*, C677T, and A1298C, each of which can decrease enzyme activity (Berk et al., 2011; Rai, 2017), especially in homozygous individuals (i.e., carry two copies). The C677T SNP polymorphism has been associated with autism in Caucasian and Asian populations (Rai, 2016).

Besides genetics, methylation activity is highly sensitive to antioxidant status (García-Giménez et al., 2017), in part because the B12 cofactor in MS is very easily oxidized. When mitochondrial aerobic metabolism is high, the production of reactive oxygen by-products can exceed the available amount of antioxidant, leading to a condition of oxidative stress (inadequate antioxidant capacity). Glutathione (GSH) is the primary intracellular antioxidant for all cells and its formation has been linked to MS activity. When vitamin B12 gets oxidized during oxidative stress, the lower MS activity diverts more HCY to increase GSH synthesis, helping to resolve the oxidative stress. In this manner vitamin B12 serves as a sensor of antioxidant status and helps to replenish it as needed (Herrmann et al., 2001). When oxidative stress turns MS off, methylation activity (including DNA methylation), is decreased and consequential changes in gene expression can also help to resolve the oxidative stress.

The GI microbiome can exert an important influence over systemic antioxidant status (Wang et al., 2017; Bonfili et al., 2018). A study examined metabolic differences between germ-free and conventionally raised mice and found that the presence or absence of a microbiome altered gene expression in the host GI tissues (Mardinoglu et al., 2015). Specifically, it was found that there was a significantly lower expression of genes involved in the synthesis of GSH from cysteine in GI tissues of conventionally raised mice, especially in the ileum, and this decrease was associated with lower absorption of a number of amino acids, including methionine. Absorption of the sulfur amino acid cysteine and its oxidized form cysteine is crucial to supply the body with the material to synthesize GSH. It is noteworthy that the cysteine transporter EAAT3 (excitatory amino acid transporter 3) is most highly expressed in the ileum, especially the distal ileum, the site of vitamin B12 absorption (Iwanaga et al., 2005; Aoyama and Nakaki, 2013; Aoyama and Nakaki, 2015; Bjørn-Yoshimoto and Underhill, 2016). This co-localization facilitates postnatal epigenetic programming in response to the level of antioxidant (Waly et al., 2012).

A number of studies have reported the presence of oxidative stress in autism, associated with a decreased plasma level of GSH (Ghanizadeh et al., 2012; Gu et al., 2015; El-Ansary et al., 2017). GSH is one of the most important detoxifying agents and is composed of three amino acids, cysteine, glycine, and glutamate (Awasthi et al., 2017; McBean, 2017). The highest concentration of GSH is in the liver, whereas brain levels are low, and depletion of glutathione leads to oxidative stress. Oxidative stress occurs when there is an imbalance between the production of reactive oxygen and a biological system’s ability to detoxify or repair oxidative damage. Children with autism have shown to have increased oxidative stress and impaired DNA methylation capacity (James et al., 2004; Frustaci et al., 2012). Indeed, the levels of oxidative stress and impaired methylation can identify autistic vs. neurotypical subjects with an accuracy of 97% (Howsmon et al., 2017).

### Gastrointestinal Distress

Among other complications, gastrointestinal problems are by far the most common and GI disease is more prevalent in individuals with autism (Hsiao, 2014; Isaksson et al., 2017; Holingue et al., 2018). GI symptoms occur in about half of children with ASD, and the prevalence increases as children get older (Chaidez et al., 2014). Moreover, the severity of autism symptoms is related to the occurrence of problems in the gastrointestinal tract (Wang et al., 2011). Reported problems included chronic constipation, chronic diarrhea, abdominal pain, gastroesophageal reflux disease (GERD), gas and bloating. A multicenter study of over 14,000 individuals with ASD revealed a higher prevalence of inflammatory bowel disease (IBD) and other GI disorders in patients with ASD as compared to controls (Kohane et al., 2012). The digestive tract of children with autism revealed substantial differences compared to neuro-typical children including abnormal intestinal permeability, inflammation and different composition of intestinal microbes.

### Abnormal Intestinal Permeability

**FIGURE 4:**
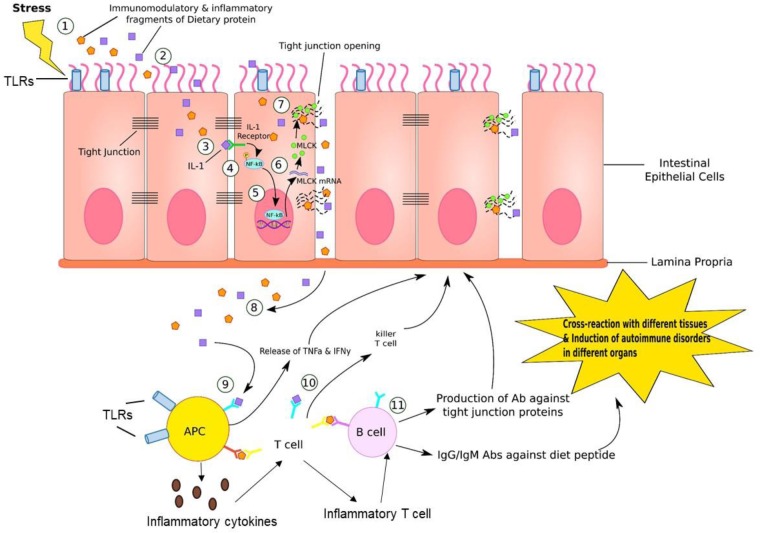
Gut–brain inflammation. (1) Stress, such as medications, neurotransmitters, enzymes, neuropeptides, intestinal flora, or immune dysregulation generates immunomodulatory and inflammatory fragments of dietary proteins. (2) These fragments can diffuse into endothelial cells lining the GI tract. (3) IL-1, which is one of the product of fragment of dietary proteins bind to IL-1 receptor on the lateral border of adjacent epithelial cell. (4) This IL-1/IL-1 receptor complex phosphorylates NF-kB. (5) Activated NF-kB further binds to DNA sequence in nucleus of endothelial cell, inducing transcription of MLCK (myosin-light chain kinase) mRNA. (6) MLCK mRNA travels to cytosol and is translated into MLCK proteins. (7) MLCK proteins bind to and open up the tight junction, where dietary fragment proteins are released into paracellular space. (8) These particles are further released into reticular tissue. (9) APC recognizes this dietary fragment and presents to T cells. (10) T cells generate killer T cell attacking epithelial cells that contain these inflammatory dietary fragments. (11) B cells are activated by T cells presenting the dietary fragment. In response, B cells generate antibodies against tight junction proteins, IgG and IgM antibodies against diet peptides. This leads to cross-reaction in various tissues and induction of autoimmune disorders in different organs. In addition, antigen-presenting cells (APC) such as dendritic cells (DCs) can produce proinflammatory cytokines that educate naive CD4+ T cells into inflammatory T cells that can help B cell maturation to produce antibodies.

While studies have shown inconsistent findings (Kushak et al., 2016), children with autism may exhibit abnormal intestinal permeability referred to as “leaky gut syndrome” (D’Eufemia et al., 1996; Turck and Michaud, 2008; de Magistris et al., 2010) (**Figure 4**). In case of a leaky gut, the defensive barrier to prevent substances in the GI tract from entering into the blood stream is compromised and is therefore referred to as “leaky” (Liu et al., 2005). With loss of the usual barrier of the gut lining, nutrient absorption can be compromised and toxins are able to enter into the blood stream. Abnormal intestinal permeability/leaky gut, is increasingly recognized as an important contributor for many different conditions, including autism, demonstrated in experimental animal models as well as in human subjects (Hsiao et al., 2013; Buscarinu et al., 2018). Increased gut permeability as indicated by increased fluorescein isothiocyanate-labeled dextran (FITC-dextran) levels in the plasma following oral gavage was observed in a BTBR mouse model of autism (Coretti et al., 2017). In agreement with these results, significantly decreased mRNA levels of occludin and zonulin was observed in male BTBR mice with a similar pattern in female mice although the levels were not significant compared to prosocial C57BL/6j (C57) mice. Occludin and zonulin are tight junction proteins that help in maintaining gut integrity (Wang et al., 2000; Weber, 2012; Sturgeon et al., 2017). In addition, gut dysbiosis, behavior alterations (demonstrated via differences in three chamber paradigm, marble burying and spontaneous self-grooming tests) and increased mRNA levels of proinflammatory markers such as CD11c, IL-10, IL-6, and TNF-alpha were observed in both sexes of BTBR mice compared to C57 mice. *Bacteroides*, *Parabacteroides*, *Sutterella*, *Dehalobacterium*, and *Oscillospira* genera, based on phylotypic evidence identified by applying the metagenomic biomarker discovery approach of LEfSe [linear discriminant analysis (LDA) effect size (LEfSe) method], were suggested to be associated with the pathological traits observed in this BTBR mouse model of autism (Coretti et al., 2017). This BTBR mouse model can serve as a valuable tool to understand the role of microbiota in interaction of gut–brain axis during autism as well as to develop novel treatment modalities based on alteration of gut microbiota.

Besides experimental animal models, altered intestinal permeability has been observed in human autism patients. A study reported intestinal permeability in 9 out of 21 autistic patients (43%) but in none of the 40 control subjects (D’Eufemia et al., 1996). In this study patients’ ages ranged from 4 to 16 years comprising 15 males and 6 females. Another study reported a high percentage of abnormal intestinal permeability values among patients with autism (36.7%) and their first-degree relatives (21.2%) compared with normal subjects (4.8%). Patients with autism on a reported gluten-casein free diet had significantly lower intestinal permeability (de Magistris et al., 2010). Further studies using larger number of autism subjects are warranted to understand how abnormal intestinal permeability/leaky gut leads to altered behavioral manifestations observed in autism. These future investigations should also take into account the differences between childhood and adulthood manifestations of abnormal intestinal permeability/leaky gut and their contribution to the development of ASD or its severity.

### Inflammation

Maternal immune activation (inflammation) can contribute to behavioral abnormalities associated with neurodevelopment in both primate and rodent offspring (Smith et al., 2007; Malkova et al., 2012; Bauman et al., 2014; Machado et al., 2015). The exposure of fetuses to maternal inflammation thus increases the likelihood of developing autism in humans. However, further clinical studies using large cohorts of autism and control subjects are warranted to decipher the precise contribution of maternal inflammation in predisposition to ASD.

Inflammation has been observed in autism patients. It was shown that 48 out of 52 (92.3%) children with regressive autism exhibited inflammation in the upper and/or lower GI tract, and intestinal biopsies in children with regressive autism indicated a novel lymphocytic enterocolitis with autoimmune features (Ashwood et al., 2003). The incidence and prevalence of pediatric IBD in the United States are estimated at 10 per 100,000 (0.01%) and 100–200 per 100,000 (10–20%) respectively (Rosen et al., 2015). The causes for gut inflammation are complex and multiple, but, in general, the same factors that cause leaky gut can trigger inflammation and vice versa. A lack of diverse gut bacteria was indicated by a study in which stool samples of children with autism and GI issues showed distinct and less diverse gut microbial composition, specifically a reduced abundance in the genera *Prevotella*, *Coprococcus*, and unclassified Veillonellaceae (Kang et al., 2013). In fact, the severity of the autistic characteristics correlated with the diversity and prevalence of some specific gut microbes such as *Firmicutes* spp.

### Different Intestinal Microbes and Gut Flora

A number of studies have demonstrated that gut microbe composition is altered in autism subjects compared to normal individuals (Parracho et al., 2005; Finegold et al., 2010; Strati et al., 2017; Kang et al., 2018). A study demonstrated that autistic subjects had significantly lower amounts of three bacteria, *Prevotella*, *Coprococcus*, and *Veillonellaceae* (Kang et al., 2013). The higher levels of *Clostridium*, *Bacteroides*, *Desulfovibrio*, *Caloramator*, and *Sarcina* have also been observed in ASD patients compared to normal control individuals (Finegold et al., 2002, 2010; Adams et al., 2011; Finegold, 2011; De Angelis et al., 2013). A relative lower abundance of *Feacalibacterium prausnitzii* and *Haemophilus parainfluenzae* has been observed in feces of children with ASD compared to neurotypical controls. A study showed a significant increase in the *Firmicutes*/*Bacteroidetes* ratio in autism patients due to a reduction of the *Bacteroidetes* relative abundance (Strati et al., 2017). At the genus level, there was a decrease in the relative abundance of *Alistipes*, *Bilophila*, *Dialister*, *Parabacteroides*, and *Veillonella* whereas there was significant increase in the prevalence of *Collinsella*, *Corynebacterium*, *Dorea*, and *Lactobacillus* in autistic subjects. Further, there was abundance of bacterial taxa belonging to *Escherichia*/*Shigella* and *Clostridium cluster* XVIII in constipated autistic individuals. The prevalence of the fungal genus Candida was more than double in the autistic than neurotypical subjects. However, this difference in fungal numbers was only partially significant between autistic and neurotypical subjects.

Besides above-mentioned microbes, *Sutterella*, a genus of anaerobic Gram-negative bacteria within the *Proteobacteria phylum*, has also been implicated in the pathophysiology of ASD. The biopsies taken from the GI tract of ASD children with GI disturbances demonstrated significantly higher prevalence of *Sutterella* species compared to control group (Williams et al., 2012). Another study also showed increased prevalence of *Sutterella* species as well as *Ruminococcus torques*, in the feces of children with ASD as compared to control group (Kang et al., 2013). Further still, a 2012 study showed higher concentrations of short chain fatty acids and ammonia in stool samples ASD children as compared with controls, suggesting altered fermentation processes and utilization of fermentation products in children with ASD (Wang et al., 2012). Although these studies clearly demonstrate that gut microbiota composition is altered in ASD patients, the body of literature in this space is still nascent and other studies suggest that the differences in the microbiomes of neurotypical children and children with ASD are either only partially significant or inconclusive (Gondalia et al., 2012; Son et al., 2015). Further studies using larger sample sizes and microbial detection techniques that are more sensitive in differentiating bacteria at the species level, are required to understand the precise contribution of this altered microbiota in predisposition to ASD.

## Antibiotic Induced Shifts in the Microbiome

One of the factors responsible for the alteration of microbe composition as discussed above is the use of antibiotics. Antibiotics significantly shift the structure of the microbial community (Dethlefsen et al., 2008; Simon et al., 2015), changing the metabolic status of the gut (Ponziani et al., 2016). Some of the changes caused by antibiotics are transient and can be reversed at the end of the treatment, while others seem irreversible. Most importantly, it has been observed that gut bacteria present a lower capacity to produce proteins, as well as display deficiencies in key activities, during and after the antibiotic treatment. For instance, antibiotics decrease the ability to absorb iron, to digest certain foods and to produce essential molecules (Pérez-Cobas et al., 2013). Previously it was assumed that short-term antibiotic treatment would alter gut microbe composition only for a short time, however, this is not the case (Jernberg et al., 2010). Even a relatively short course of antibiotics can lead to alteration in gut microbiota, which in turn can lead to severe consequences such as inflammation, immune dysregulation, allergies, infections, cardiovascular diseases, diabetes, metabolic issues, GI disease such as Crohn’s, IBD, yeast overgrowth, chronic constipation and diarrhea (Jernberg et al., 2007; Yang et al., 2009; Ubeda et al., 2010; Sobhani et al., 2011; Buffie et al., 2012; Rutten et al., 2015; Lau et al., 2017; Ni et al., 2017).

In 2010 there were nearly 23 million courses of Amoxicillin or Augmentin prescribed to children in the United States and more than 6.5 million of those courses were for children under the age of two (Chai et al., 2012; Blaser, 2014). Studies have shown that children with autism have had significantly more ear infections than control groups, leading to more antibiotic prescriptions (Niehus and Lord, 2006; Adams et al., 2016; Leung and Wong, 2017). Furthermore, another study showed that 34.5% of children with autism had used extensive and repeated broad-spectrum antibiotic treatments (>6 courses) compared to control group (0% with more than 6 courses) (Parracho et al., 2005). In fact, a higher proportion of 54.5% of the children with autism had received more than six courses of antibiotics suggesting frequent over-prescription of antibiotics. Further prospective studies are warranted to understand how the overuse of antibiotics in the 1st years of life might disrupt the gut–brain axis and lead to the development of future neurological disorders including autism.

## Microbiome and Antibiotic Use in Infancy as Well as Early Childhood

A baby’s first exposure to the natural microbial world occurs during vaginal delivery (Martin et al., 2016). A woman’s vaginal flora carries lactobacilli bacteria, which make the vaginal canal more acidic, a milieu that helps in establishing defense against pathogenic bacteria (Collado et al., 2016). In contrast, cesarean delivery is associated with long-term differences in the intestinal flora (Neu and Rushing, 2011). The primary gut flora in infants born via C-section is disturbed for up to 6 months after birth (Grönlund et al., 1999; Biasucci et al., 2008), and children born via C-section have less protection against pathogenic invaders than infants born via vaginal delivery (Huurre et al., 2008; Biasucci et al., 2010). Currently the CDC estimates that more than 32% of babies in the United States are born via C-section (Centers for Disease Control and Prevention [CDC], 2014).

The first microbes that colonize the infants gut set the stage for a more adult-like microbiota in later years. Research has confirmed that delivery mode shapes the acquisition and structure of the initial microbiota of newborns (Biasucci et al., 2008) (**Figure 1**). Infants born via vaginal delivery displayed bacterial communities resembling their own mother’s vaginal microbiota, dominated by *Lactobacillus*, *Prevotella*, or *Sneathia* spp., while babies born via C-section harbored bacterial communities similar to those found on the skin surface, dominated by *Staphylococcus*, *Corynebacterium*, and *Propionibacterium* spp. This finding would explain in part why babies born via C-section are more vulnerable to certain pathogens, making them more susceptible to infections. It is estimated that 64 to 82% of reported cases of methicillin-resistant *Staphylococcus aureus* (MRSA) skin infections in newborns occurred in Cesarean-delivered infants (Centers for Disease Control and Prevention [CDC], 2006).

After birth, nutrition and environment play crucial roles in determining the baby’s microbial constitution. Right after birth the baby instinctively reaches for the mother’s nipple, bringing together the lactobacilli from the birth process in contact with the milk. Lactobacilli and other lactic acid producing bacteria break down lactose, the sugar in milk that converts into energy. Also, for the first few days after birth, the breasts of the mother produce colostrum. Colostrum contains antibodies to protect the newborn against disease, and contains less fat but delivers more protein than mature milk. The immune-protective properties of colostrum are crucial for early life. It contains numerous antibodies called “secretory immunoglobulin” (IgA) that help protect mucous membranes in the throat, lungs, and intestines. Leukocytes are also present in large numbers, which begin protecting the infant from harmful viruses and bacteria. Colostrum further enhances the healthy gut bacterial constitution by providing more beneficial bacteria. Since the newborn’s digestive tract is still very immature, colostrum delivers its nutrients in a very concentrated low-volume form; its mild laxative effect encourages passing of the baby’s first stool (Godhia and Patel, 2013).

The lactating mother’s milk microbiome changes and varies according to the delivery mode and the maternal weight. Breast milk from women who gave birth via cesarean section is less diverse in bacterial composition than the breast milk in mothers who gave birth via vaginal delivery (Urbaniak et al., 2016). This discrepancy suggests that hormonal signals during the vaginal birth process may influence the diversity of microbes in breast milk (Cabrera-Rubio et al., 2012). As soon as milk production begins, the nursing baby will continue to receive the mother’s microbes, allowing the mother’s beneficial gut bacteria to be directly transferred to the neonate’s gut via her breast milk. Indeed, the same strains of *Bifidobacterium breve* and several types of *Clostridium* bacteria are present in neonates as the mother (Jost et al., 2014).

In addition to above discussed factors, antibiotics can also play a crucial role in changes in the gut microbiome. The age of first exposure to antibiotics is very important for the future overall health of many individuals. Early colonization of healthy, beneficial gut bacteria is vitally important to maintain gut homeostasis, but the use of antibiotics during infancy can drastically alter the microbiome. Young children are the most vulnerable to the use and over-use of antibiotics. By the age of three, 80% of children have suffered at least one acute infection of the middle ear for which antibiotics are prescribed and more than 40% of children experience at least six of these acute infections of the middle ear by age seven (Blaser, 2014).

At birth the brain is only 25% wired, by the age of one it is about 75% wired and by the age of three the brain is wired up to 90% (Chédotal and Richards, 2010). At the same time, microbial colonization is actively taking place, setting the stage for future digestive health outcomes, as well as mental health and well-being. Only recently have we begun to understand the role of the early-life gut microbiota in the development of immune-mediated, metabolic, and neurological diseases. The human microbiome develops from birth until about the age of three, and antibiotics use during these formative years can disrupt the process (Arrieta et al., 2014). Recent research has shown that the immune system of infants is also in development during early years and not inborn as previously assumed (Simon et al., 2015). In a twin study, researchers dispelled the belief that the body’s immune system is genetically programmed (Brodin et al., 2015). Since gut microbes regulate the immune system, antibiotic use in the 1st years of life can crucially impact maturation of the immune system. Further investigations using larger cohorts are warranted to understand how the antibiotic usage during infancy and early childhood leads to alterations in gut microbiota affecting gut–brain axis and hence, may increase predisposition to ASD.

## Gut – Brain Connection and the Blood–Brain Barrier

**FIGURE 5:**
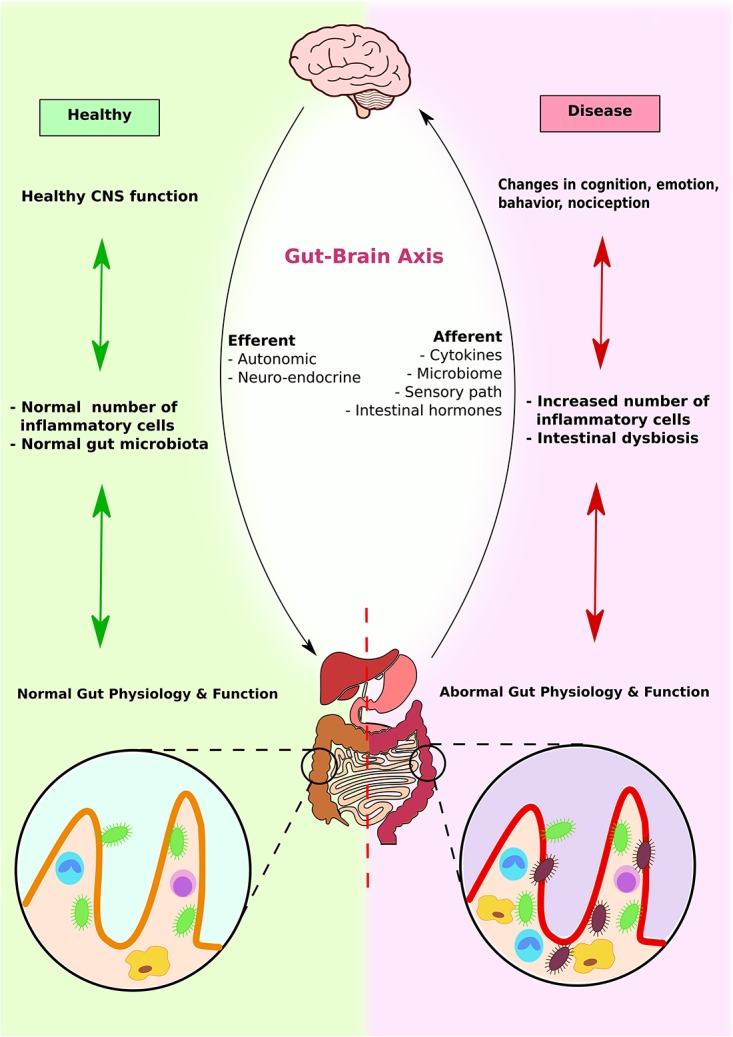
Gut–brain axis and microbiota interplay. Brain, GI system, and microbiota interact with each other to produce physiological responses. In healthy individuals, CNS function enhances normal immune response, which promotes colonization of normal gut microbiota and maximizes GI function. In contrast, in diseased individuals, altered brain functions induce abnormal immune response and intestinal dysbiosis. This further contributes to abnormal gut physiology and function.

As discussed earlier, the enteric nervous system provides bidirectional communication between gastrointestinal cells and the central nervous system (Mittal et al., 2017) (**Figure 5**). Moreover, gut microbiome alterations can unbalance gastrointestinal immune responses and influence distal effector sites, leading to CNS disease including demyelination and affective disorders (Ochoa-Repáraz and Kasper, 2014; Moya-Pérez et al., 2017). Serotonin and inflammation can have a significant influence on the functioning of the gut–brain connection. In addition, gut microbes can also influence the integrity of blood–brain barrier as discussed below.

### Serotonin: The Neurotransmitter in the Gut

Serotonin is a neurotransmitter regulating many functions of the human body, such as mood, sleep, appetite, temperature regulation, learning and memory and social behavior (Blanchard and Meyza, 2017; Brummelte et al., 2017; Gasparini et al., 2017). Serotonin is also involved in various functions of the cardiovascular, musculoskeletal and endocrine systems (Young, 2007; Elliott et al., 2008; Ayme-Dietrich et al., 2017; Shively et al., 2017). A deficiency in serotonin levels leads to many symptoms that most individuals with autism exhibit, such as anxiety, poor sleep, inability to focus, agitation, mood swings, and depression (Whitaker-Azmitia, 2001; Olivier, 2015; Blanchard and Meyza, 2017).

While 90% of serotonin is produced in the gut, it is also released in the CNS, especially midbrain, hypothalamus, limbic system, cerebellum, pineal gland, and spinal cord (Marieb and Hoen, 2007). It has been suggested that gut bacteria can play a crucial role in the production of serotonin (Reigstad et al., 2015; Hata et al., 2017). A study compared serotonin levels in germ-free mice to mice with gut microbes, and found that the germ-free mice produced significantly less serotonin (Yano et al., 2015). These results indicate that gut microbiota can be important determinants of enteric serotonin production and homeostasis.

Tryptophan is the metabolic precursor for serotonin, niacin (vitamin B-3) and picolinic acid and is needed for normal growth in infants and for nitrogen balance in adults. It is an essential amino acid, and must be obtained via food (Murray, 2013; United States Department of Health and Human Services, 2016). Tryptophan metabolism has shown to be reduced in patients with autism (Boccuto et al., 2013) and the commensal bacterium, *Bifidobacteria infantis*, which is a probiotic, has been shown to be involved in tryptophan metabolism in rat model (Desbonnet et al., 2008; Bravoa et al., 2011).

### Inflammation and the Gut–Brain Connection

Besides serotonin, inflammation can influence the gut–brain axis. Inflammation is characterized by the production of pro-inflammatory cytokines such as tumor necrosis factor alpha (TNF-α). TNF-α levels are elevated in autism (Chez et al., 2007), and brains of autistic individuals display a pattern of elevated immune response, including activation of microglial cells, whose function is to eliminate pathogens and other threats (Gupta et al., 2014). Microglia are glial cells functioning as resident macrophages of the brain and the spinal cord, providing the primary active immune defense in the central nervous system (CNS).

Since 70–80% of human immune cells are located in gut-associated lymphoid tissue, lymphocyte accumulation and differentiation in the gastrointestinal tract can be triggered in response to changes in microbiota composition (Yamanaka et al., 2003; Douglas-Escobar et al., 2013). Mucosal surfaces of the intestinal tract are continuously exposed to both pathogenic and beneficial microorganisms, and these gut mucosal cells can trigger either pro- or anti-inflammatory responses. Gut epithelial cells express toll-like receptors (TLRs) that can help identify and differentiate between beneficial and pathogenic bacteria, making them crucial for maintaining gut homeostasis (Rakoff-Nahoum et al., 2004). Acute mucosal inflammation due to enteric bacterial pathogens can cause the chronic inflammatory response, but the events linking inflammatory activation in the gut to activation of glial cells and microglia in the brain requires further investigation. If inflammation in the gut lining causes gut permeability or leaky gut, pathogenic bacteria can escape through the gut lining into the bloodstream, and inflammatory cytokines traveling through the bloodstream can cause oxidative stress and promote systemic immune responses (Clayburgh et al., 2004).

### The Blood–Brain-Barrier

**FIGURE 6:**
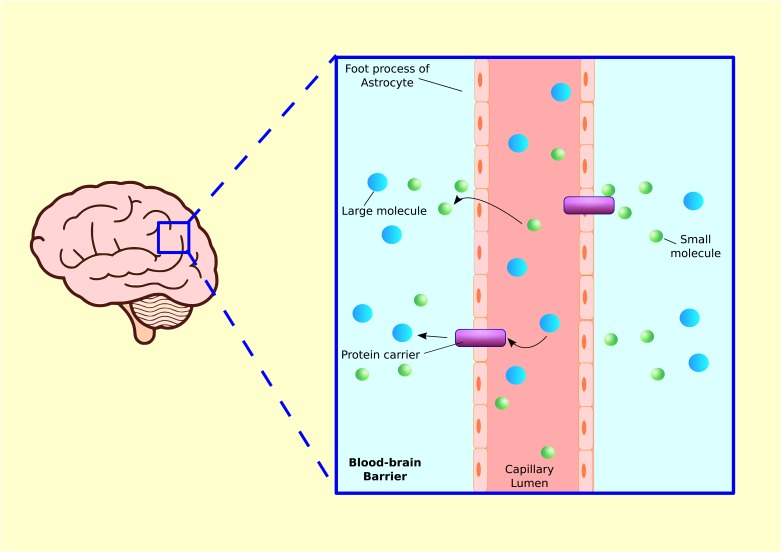
Blood–brain barrier. Blood capillaries are surrounded by astrocyte processes, which enhance transcapillary molecular transport. Small molecules such as gasses or lipid soluble substances in capillary lumen can travel into tissue fluid via diffusion. Larger molecules such as glucose, amino acid, or other hydrophilic proteins are released from brain capillary into tissue via protein carriers.

Just as the gut has an epithelial lining that prevents pathogens from entering the blood stream, the brain also has a protective barrier to keep foreign invaders from entering the brain (Ballabh et al., 2004; Blanchette and Daneman, 2015). The blood–brain barrier (BBB) is a layer of tightly packed endothelial cells that make up the walls of brain capillaries. The primary function of the BBB is to prevent free diffusion of substances from the blood into the brain and CSF. The passage across the membrane is selective, by means of lipid bilayer solubility and/or recognition by select transport molecules (Engelhardt and Sorokin, 2009) (**Figure 6**). Endothelial cells inhibit the diffusion of microscopic substances such as bacteria and large or hydrophilic molecules into the CSF, while allowing the diffusion of small hydrophobic molecules (Obermeier et al., 2016). Earlier it was believed that the BBB is very difficult to penetrate, even in a newborn, and that the brain is fiercely protected from bacteria and viruses. However, this notion is beginning to fade as the effects of inflammation on the BBB are better understood. It is now believed that the permeability properties of the BBB are not fixed, and inflammation is one of the important factors impacting the BBB permeability (de Wit et al., 2016). Induction of an inflammatory response in mice via LPS injection caused a long-term increase in BBB permeability (Stolp et al., 2009, 2011). Thus, an inflammatory insult during brain development can change BBB permeability and alter behavior in later life.

Gut microbes have been linked to altered BBB development and function during neurological disorders (Mirza and Mao-Draayer, 2017), and a study in mice showed that gut microbes influence BBB penetrability (Bravoa et al., 2011). The development of the BBB between germ-free fetal mice and those with normal microbes was compared. It was observed that maternal gut microbes in late pregnancy can be neuroprotective and can influence BBB permeability in the offspring. Interestingly, the mice with normal microbes developed a strong BBB with tight junctions toward the late stages of fetal development, preventing entry of a tracer antibody. In fetuses whose mothers were germ-free, however, the antibody continued to enter the brain tissue, even late in pregnancy, demonstrating that stability of the BBB in the fetus depends upon the mother’s microbial flora (Bravoa et al., 2011).

Even after birth, the BBB is not a fixed entity and can still be influenced by gut microbes. When germ-free adult mice underwent fecal transplantation from animals with normal microbiomes, the junction proteins tightened, resulting in decreased BBB permeability (Saunders et al., 2012). These results suggest that dysbiosis in the GI tract can have adverse effects on BBB permeability.

## Mitochondria Dysfunction and Its Connection to Oxidative Stress

Besides normal functioning of the brain and intact BBB, the health of the cell depends on the proper functioning of mitochondria, which generate energy and adenosine triphosphate (ATP) (Kang et al., 2017). Mitochondria carry their own DNA, which codes for 37 proteins, 13 of which are subunits of oxidative phosphorylation which is crucial for the formation of ATP (Wang et al., 2004; Taylor and Turnbull, 2005). The ability of the mitochondria to generate energy is especially important for proper functioning of the central nervous system since brain cells require a lot of energy to communicate with each other. As a proof of this concept, mitochondrial dysfunction has been implicated in many neurological and psychiatric diseases, including neurodegenerative diseases (Verity et al., 2010; Frye and Rossignol, 2011; Ganguly et al., 2017; Gao et al., 2017; Smith et al., 2017). Clinical findings confirm that a significant subset of children with autism suffer from underlying mitochondrial dysfunction (Giulivi et al., 2010; Griffiths and Levy, 2017; Hollis et al., 2017). Mitochondria in granulocytes of children with autism consume far less oxygen than those of typically developing children, which is an indication of mitochondrial dysfunction (Napoli et al., 2014).

Mounting evidence shows that certain antibiotics can cause mitochondrial dysfunction. In addition to depleting the microbiota and altering immune function in the gut, antibiotics damage intestinal epithelium, a major insult to proper functioning in nutrient absorption and immune system regulation (Morgun et al., 2015). This study found that antibiotics and antibiotic-resistant microbes induced repression of genes coding for proteins constituting all five complexes of the mitochondrial respiratory chain. This significant finding was confirmed by another study where it was demonstrated that clinically relevant levels of antibiotics can cause mitochondrial dysfunction and lead to the production of detrimental reactive oxygen species (ROS) in mammalian cells. This was evident both *in vitro* and *in vivo* studies (Kalghatgi et al., 2013). ROS can directly interact with cellular components resulting in DNA, protein, and lipid damage. Several antibiotics, specifically tetracycline, minocycline, chloramphenicol and aminoglycosides are suspected to be “mito-toxic,” because they inhibit mitochondrial DNA translation and protein synthesis (Balcells, 2016; Morén et al., 2016).

In mitochondrial dysfunction, cells cannot generate sufficient energy which ultimately can lead to apoptosis. The interconnectivity among mitochondrial dysfunction, oxidative stress and inflammation becomes evident, and all three are commonly observed in individuals with autism (Rossignol and Frye, 2014). In addition, environmental factors such as pesticides, cigarette smoke and radiation all can contribute to mitochondrial dysfunction (Kam and Banati, 2013; Meyer et al., 2013). The vulnerability of mitochondria to broad environmental toxins may be partially due to the fact that mitochondria have a negative potential and alkaline pH in the matrix, and that mitochondrial membranes have high lipid content, these properties make them accumulate cationic metals, amphiphilic organic chemicals and lipophilic compounds, leading to mitochondrial dysfunction and increased susceptibility to neurological diseases due to energy depletion.

## Environment and the Epigenetic Connection in Autism

In addition to above discussed factors, the environment can play a crucial role in predisposition to ASD (Tran and Miyake, 2017; Waye and Cheng, 2018). The sharp rise in autism diagnosis in recent years leaves little doubt that autism cannot be purely genetic, however, we should examine the interactions between genes and the environment and particularly the role of epigenetics (Moosa et al., 2017; Siu and Weksberg, 2017; Eshraghi et al., 2018). Recognition of the role of the GI tract in cysteine absorption, and GSH production, and its influence over DNA and histone methylation, provides a novel perspective on how the microbiome and the use of antibiotics can exert effects on development, especially brain development.

Epigenetic regulation of gene expression is tied to chemical modifications (e.g., the addition of methyl groups) to DNA and to the histone proteins that associate tightly with DNA in the nucleus (Nardone et al., 2017; Roubroeks et al., 2017). These dynamic modifications can determine when or even if a given gene is expressed in a cell or organism. The science of epigenetics is gaining widespread attention as scientists are learning more about the complexities of how environment and lifestyle influences on DNA expression are bringing about epigenetic changes which can last a lifetime or even be transmitted across generations via germline cells (Wątroba et al., 2017; Virzì et al., 2018). Until recently, medical science primarily attributed disease to genetic determinism, but the more recent concept is introducing the causes for many diseases as epigenetic triggers, especially when certain diseases are more prevalent in specific areas or when the incidence of a disease dramatically increases.

There is a need to understand which environmental factors are combining with genetic susceptibility to increase autism prevalence. Genetics are unquestionably involved, however, genetic susceptibility and exposure to certain environmental insults that can trigger epigenetic changes provides a more reasonable understanding of autism (Waye and Cheng, 2018). An autism twin study concluded that there is considerable monozygotic (MZ) twin discordance, indicating a significant role for non-genetic factors (Wong et al., 2014). Since monozygotic twins share the same DNA sequence, differences in autism traits imply epigenetic involvement. Gene expression data in autism provide evidence for abnormalities in peripheral blood leukocytes that could represent a genetic and/or environmental predisposition to the disorder (Gregg et al., 2008).

It has been demonstrated that specific variants of the *NOD2* gene that carry a high risk of developing IBD to their carriers are also associated with an altered intestinal microbiome (Naser et al., 2012). This study focused on the genes shaping the types of microbes that reside in the human gut, however, it would also be interesting to investigate if the human microbiome can alter gene expression, given that our microbial composition is more flexible and variable than our genes. Our diet has a profound impact and causes changes in our microbiome (David et al., 2014). Since the environment and food can alter the human microbiota, it is probable that there is a direct and complex interaction between human genes and microbiota, our second genome that needs to be explored in future investigations.

Transgenerational epigenetic effects are defined as effects on the phenotype (or on patterns of gene expression) that are detected across more than one generation and that cannot be explained by changes to the primary DNA sequence (Gröger et al., 2016; Pilling et al., 2017; Sharma, 2017). This includes epigenetic effects of environmental exposures on adults that alter the phenotype of the developing embryo via the placenta or the newborn via the milk (Daxinger and Whitelaw, 2012). A good example of transgenerational epigenetic consequences is BPA (bisphenol A) exposure that has shown to affect fertility in mice for three generations (Ziv-Gal et al., 2015). Pregnant mice exposed to low-dose BPA had significantly higher fertility and reproduction problems for three generations, as compared to the control group.

There is some compelling evidence that the microbial composition is directly responsible for triggering epigenetic changes. For example, a Japanese study showed that butyrate (a histone deacetylase inhibitor), a by-product of the digestion of dietary fiber by gut microbes, acts as an epigenetic switch that boosts the immune system by inducing production of regulatory T cells in the colon (Furusawa et al., 2013). One can hypothesize that changes in microbiota, especially in the early stages of life, could directly be responsible for turning on or off certain genes, in this case early use and overuse of antibiotics causing a shift in the microbial diversity and may be turning on the autism gene. Further studies are warranted to confirm this hypothesis that will shed light on the interconnection between epigenetics and alteration in gut microbiome.

## Conclusion and Future Directions

The microbiome is responsible for many functions that are impaired in autism such as metabolizing food, regulating the immune system, eliminating toxins and waste, absorbing nutrients, producing neurotransmitters, preventing the colonization of the gut by pathogenic bacteria, and maintaining the tight junctions of intestinal epithelial cells. Microbial constitution and development in early childhood has been shown to affect the blood–brain barrier permeability. Early use and over-use of antibiotics can lead to an imbalance between beneficial microbes and pathogenic microbes, which can in turn lead to inflammation, immune dysregulation, allergies, diabetes, metabolic problems, yeast overgrowth, and gastrointestinal complications. Not surprisingly, all of these pathologies display an increased incidence in children with autism. Although GI complications have been associated with ASD, the precise prevalence of these complications is still not known. The reported estimates of GI symptoms in ASD subjects vary widely from 9–91% (Buie et al., 2010; Fulceri et al., 2016). These very large variations can be attributed to small sample size, different methodological approaches (e.g., data source and time period for reporting), different study populations and lack of consensus in the clinicians regarding GI symptomology. Further studies using large cohorts and consensus in clinicians about GI symptomology is warranted in order to precisely estimate the prevalence of GI complications in ASD subjects and how it can contribute to the development of autism.

In addition to GI symptoms, there has been a significant discrepancy regarding altered gut microbiota composition in ASD patients compared to neurotypical subjects. This discrepancy may be due to differences in technologies used to determine microbial composition, geographical differences between participants (which may result in genetic and/or dietary differences), potential sub-types of gut microbiota within ASD groups, small sample size and inadequate statistical control for testing multiple-hypotheses. Studies using large sample sizes from the same geographical area and robust statistical analysis of the data will help in addressing some of these issues. In addition, emerging technologies that involve characterizing metabolomics profiles that can be correlated with gut microbial structure and interrelated functional pathways will provide valuable information to understand the role of the gut microbiota in autism.

Besides GI complications, brain inflammation is a hallmark comorbid pathology observed in autism. Longitudinal studies of brain inflammation compared to gut inflammation and microbial imbalance needs to be performed. The inflammatory insults during the developmental stages of brain maturation can affect blood–brain barrier permeability and may lead to encephalitis. Furthermore, epigenetic dysregulation is an important consideration in the etiology of autism, reflecting the impact of food, drugs and the environment on the intestinal microbiome. Alteration of the intestinal microbiome can lead to altered genetic expression and potentially contribute to autism causation.

Through the many pathways elucidated above, the microbiome of the human gut can be seen to play an important role in the etiology of autism. This field shows promise for understanding the true pathogenesis of this increasingly prevalent disease. A deeper understanding about gut–brain axis underlying pathogenesis of autism and how alteration in gut microbiota leads to oxidative stress in ASD patients will open up novel avenues for the management, screening and prophylaxis of autism as well developing novel treatment modalities for ASD.

## Author Contributions

All authors listed have made a substantial, direct and intellectual contribution to the work, and approved it for publication.

## Conflict of Interest Statement

The authors declare that the research was conducted in the absence of any commercial or financial relationships that could be construed as a potential conflict of interest.
